# Daily Consumption of Apigenin Prevents Acute Lymphoma/Lymphoblastic Leukemia in Male C57BL/6J Mice Exposed to Space-like Radiation

**DOI:** 10.3390/cancers17213513

**Published:** 2025-10-31

**Authors:** Tanat Peanlikhit, Jingxuan Liu, Tahmeena Ahmed, James S. Welsh, Tobias Karakach, Kenneth R. Shroyer, Elbert Whorton, Kanokporn Noy Rithidech

**Affiliations:** 1 Department of Pathology, Renaissance School of Medicine, Stony Brook University, Stony Brook, NY 11794, USA; 2 Department of Pathology and Laboratory Medicine, St Luke’s University Health Network, Bethlehem, PA 18015, USA; 3 Department of Radiation Oncology, Loyola University Health System, Maywood, IL 60153, USA; 4 Department of Pharmacology, Dalhousie University, Halifax, NS B3H 4R2, Canada; 5 StatCom, P.O. Box 3041, Galveston, TX 77551, USA

**Keywords:** apigenin, space radiation, protection, mitigation, chemoprevention, lymphoblastic leukemia

## Abstract

Introduction: The work presented here is part of our study series aimed at investigating the countermeasure effectiveness of apigenin (AP) against both early and late effects of heavy silicon (^28^Si) on the same cohort of exposed male C57BL/6 mice. We previously reported the countermeasure of AP against ^28^Si-induced early effects of ^28^Si ions. This section focuses on the protective effects of AP on late effects, specifically on the induction of acute lymphoma/lymphoblastic leukemia. Method: Mice received a diet containing 20 mg/kg body weight of AP for five days before and after total-body irradiation with either 0 or 0.5 Gy of 260 MeV ^28^Si ions. They were divided into four groups based on AP intake and irradiation status. At one-week after irradiation, six mice from each group were euthanized to assess AP’s effectiveness against early inflammation (in the bone marrow and gut tissues) and gut dysbiosis. The remaining mice were monitored until approximately 770 days of age. Incidence rates were analyzed using Chi-Square tests, while survival data were evaluated with Kaplan–Meier plots and log-rank tests, setting significance at *p* ≤ 0.05. Results: At 770 days, survival rates were 37% for ^28^Si-exposed mice and 63% for those consuming AP, despite irradiation. There was a 2.57-fold increase in acute lymphoma/lymphoblastic leukemia incidence among ^28^Si-exposed mice not receiving AP compared to controls and AP-fed mice. Together with our previous report on the countermeasure activity of AP against early effects, these findings suggest that the gut–bone marrow axis plays an important role in ^28^Si-induced acute lymphoma/lymphoblastic leukemia. Conclusion: Our findings demonstrate that AP is an effective means of tackling the challenges posed by space radiation, and it has the potential to revolutionize protection in this critical area.

## Simple Summary

Cancer induction from space radiation poses a significant health risk for astronauts, necessitating effective protective measures. Current shielding is inadequate, highlighting the need for novel biological countermeasures. One promising candidate is apigenin (AP), a natural compound known for its antioxidant and anti-inflammatory effects. This study focuses on how AP might help protect mice from developing acute lymphoma/lymphoblastic leukemia after being exposed to silicon ions (^28^Si). The results showed that mice not fed an AP diet had a 2.25 times higher chance of developing cancer compared to those that did eat AP. This suggests that AP could help prevent cancer caused by radiation. We believe that AP works by reducing inflammation caused by radiation in both the bone marrow and the gut, and it may also help keep the gut bacteria healthy. We also propose that the connection between the gut microbiome and the bone marrow (called the gut–bone marrow axis) plays a role in how the immune system responds to ^28^Si and impacts the development of these cancers.

## 1. Introduction

The space environment contains various types of radiation that differ from those found in Earth’s atmosphere. These include solar particle events (SPEs) and high-energy, high-mass (HZE) ions, also known as heavy ions. As a result, astronauts are inevitably exposed to space radiation during spaceflight. Research has shown that the development of fatal cancer is considered the most significant risk associated with exposure to heavy ions in the space environment [1]. To ensure the success of space missions, astronauts must be protected from radiation (shielding) or minimize the deleterious effects of space radiation (biological countermeasures). Currently, the use of shielding materials to prevent radiation exposure in space remains inadequate [2,3], underscoring the need to develop novel biological countermeasures to mitigate the harmful effects of space radiation.

The work presented here is part of our study series aimed at investigating the effectiveness of apigenin (AP) as a countermeasure against both early and late effects of heavy silicon (^28^Si) ions on the cells and tissues of exposed male C57BL/6 mice. We gave the AP diet to mice before and after ^28^Si-irradiation to prevent and mitigate the harmful effects of ^28^Si ions. For the early effects (samples collected at d7 post-irradiation), we previously reported that daily consumption of AP diet protected bone marrow (BM) cells [4] and the gut tissue [5] from ^28^Si-ion-induced inflammation. This segment of our research focused on the benefits of daily consumption of the AP diet against the late effects of ^28^Si-exposure. Given that galactic cosmic rays include a variety of heavy ions, ^28^Si ions were recommended for our research by the National Aeronautics and Space Administration (NASA) at the beginning of this project (personal communication). We chose C57BL/6 mice as our experimental model because this strain is commonly recommended for studying medical and biological countermeasures [6]. Our primary focus was on lymphoma, particularly thymic lymphoma, as it serves as a classical model for examining radiation-induced carcinogenesis in C57BL/6 mice [7,8]. This type of lymphoma can indeed be induced by X rays with a high incidence, exceeding 50% in this mouse strain [9]. It is noteworthy that, as reviewed by Rivina and Schiestl et al. [10], historically, it has been the norm in rodent pathology toxicology and experimental studies, that the distinction between lymphoma and leukemia is often less strictly defined compared to human pathology. Consequently, we used the term acute lymphoma/lymphoblastic leukemia throughout this study. The spontaneous incidence of acute lymphoma/lymphoblastic leukemia in male C57BL/6 mice is about 7% [11], but it has also been recorded as low as 1–2% [9,12]. However, based on x-irradiation data (4 fractions of 1.7 Gy once a week), the incidence of x-ray-induced lymphoma is very high (>50%) and with a short latency (3–6 mos post-irradiation) [13]. To date, there is no information on ^28^Si-induced acute lymphoma/lymphoblastic leukemia in exposed male C57BL/6 mice. Nonetheless, lymphoma of the brain (a very uncommon cancer) has been reported in some astronauts [14].

Relating to the mitigation of radiation-induced cancer, it has been shown that nutritional antioxidants significantly reduced the risk of cancer in the A-bomb survivor population [15]. Attempts have been made to develop radiation countermeasures for space radiation [3,16]. However, no novel compounds have been identified yet, and further investigations are needed. It was found that Vitamin A strongly reduces the induction of fibroma in rats exposed to swift ^56^Fe ions [17]. Dietary supplementation with Bowan-Birk protease inhibitors (BBI) or a combination of selected antioxidant agents decreased the incidence of lymphoma [18] or rare tumors (such as Harderian gland) [19] in CBA/JRC HSD mice exposed to 0.5 Gy of 1 GeV/n ^56^Fe ions, while there is no such countermeasures for ^28^Si-induced acute lymphoma/lymphoblastic leukemia.

**Figure 1:**
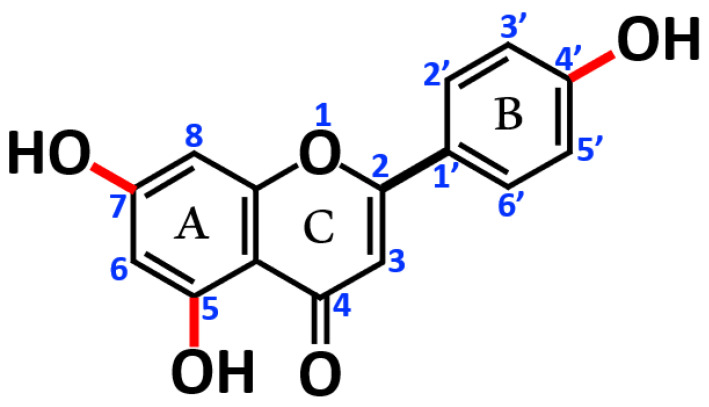
Chemical structure of apigenin.

Apigenin (AP, also known as 4′,5,7,-trihydroxy flavone or 5,7-Dihydroxy-2-(4-hydroxyphenyl)-4H-1-benzopyran-4-one) is a flavone compound with potent antioxidant, anti-inflammatory, anti-cancer, and anti-bacterial activities [20,21,22,23,24]. Figure 1 shows the chemical structure of AP. It has also been suggested that diets rich in AP or other flavones lower the risk of certain cancers, including breast, digestive tract, skin, prostate, and some blood cancers, suggesting the chemopreventive effects of AP [25,26]. Additionally, AP may help to reduce the risks of developing diseases linked to oxidative stress, e.g., cardiovascular and neurological disorders.

Results from in vitro studies show that AP effectively inhibits cancer cell growth and leads to the death of cancer cells in a dose- and time-dependent manner, via apoptosis and autophagy through the inhibition of the PI3K/Akt/mTOR and RAF/MEK/ERK pathways [22,23,26,27]. AP also inhibits MAPK including GTPase activities [28]. It has also been shown that AP inhibits phosphorylation and degradation of the inhibitor of NF-κB (IκBα) by inhibiting the activation of the IκB kinase (IKK), leading to suppression of NF-κB activation [29]. Hence, AP would be a valuable countermeasure since it will reduce levels of pro-inflammatory mediators (or cytokines, e.g., TNF-α, IL-1α, and IL-6) and oxidative damage by targeting the NF-κB pathways [4,5,30,31,32]. These inhibitory activities of AP are attributed to its capability to dock against the active site of these proteins or enzymes, resulting in the inhibition of the enzymes. Recently, we conducted molecular docking studies to assess the binding energy and interactions of AP and 16 related flavonoids with the proteins (enzymes) to identify inhibitors of these proteins and to understand how the structural configurations of flavonoids influence their binding energy [20]. We found that AP has high binding energies to all the tested proteins, indicating strong binding affinity or inhibitory potential of AP. The results from our docking studies also demonstrate that the calculated binding energy is a good surrogate for the inhibitory potential of a drug. We also found that AP possesses a better inhibitory activity than some other flavonoids, e.g., quercetin and genistein, for some specific proteins. AP has shown considerable promise as a chemopreventive and/or chemotherapeutic agent for several types of human solid tumors, including the breast, the cervix, the colon, the lung, the ovary, the prostate, and leukemia [25,33,34,35,36]. However, the majority of these studies were conducted in vitro. There are no current cancer bioassays that are comparable to our research, and clinical trials assessing the safety and efficacy of apigenin in cancer treatment remain limited. Some small-scale studies indicate that AP, as a chemopreventive and/or chemotherapeutic agent for cancer, is well-tolerated and may offer beneficial effects, but further research is necessary.

Regarding the radiation countermeasure activity of AP, we have pioneered the discovery of the beneficial effects of AP both in vitro protection [37] and in vivo mitigation [38]. Subsequently, several investigators confirmed our findings both in vitro [39,40,41] and in vivo systems [42,43]. We have demonstrated that AP counteracts harmful radiation-induced effects through its potent anti-inflammatory and antioxidative activities, as well as anti-clastogenic activity. We also found that AP increased the level of global 5-hydroxy-methyl cytosine in bone marrow (BM) cells of irradiated mice to a normal level (personal communication). Importantly, we found, for the first time, that AP can restore the balance of the gut microbiome of mice exposed whole-body to Si ions [5], reflecting one of the counteracting mechanisms of AP.

These findings establish a solid foundation for this study, which aimed to investigate the protective/mitigative potential of AP against the induction of acute lymphoma/lymphoblastic leukemia in mice exposed to ^28^Si ions. Our central hypothesis is that AP effectively exerts its chemopreventive effects by suppressing radiation-induced inflammation and preventing radiation-induced gut dysbiosis during the initial step of neoplasia.

## 2. Materials and Methods

### 2.1. Animals

All male C57BL/6J mice included in this study were part of the same cohort used to investigate the effectiveness of AP as a countermeasure against early damage in hematopoietic cells [4] and in gut tissue [5] caused by ^28^Si-irradiation in samples collected from groups mice (6 mice in each) at day 7 (d7) post-irradiation. The remaining mice in each group after the d7 sampling were monitored for morbidity and mortality until they reached approximately 770 days of age, at which point all remaining mice were euthanized.

All mice were purchased from the Jackson Laboratory (Bar Harbor, ME, USA). Following acclimatization in a specific pathogen-free barrier facility at the Division of Laboratory Animal Resources (DLAR), the mice were housed individually until they reached 10 to 12 weeks of age for ^28^Si-irradiation. Before the experiments, all mice were assigned identification codes and randomly selected for inclusion in each experimental group.

### 2.2. AP Diet Supplement

**Figure 2:**
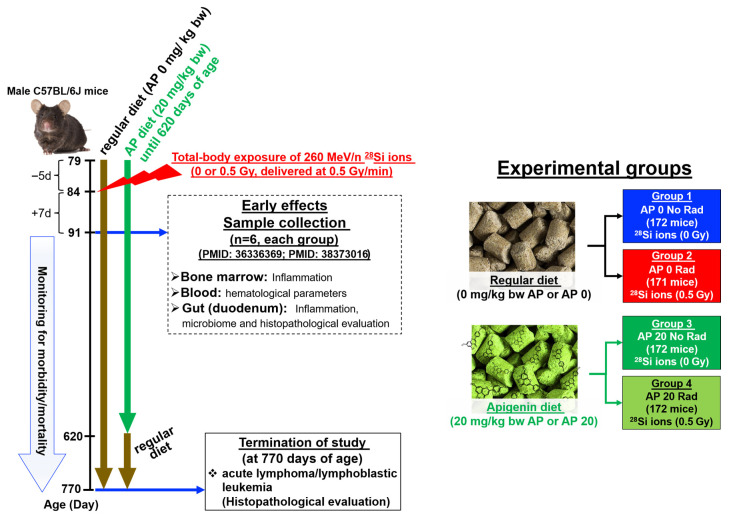
Experimental design.

Apigenin (CAS 52—36-5, 99% purity, commercially available) was purchased from AdooQBioscinece (Irvine, CA, USA). Rodent diet #5053 (purchased from Lab Supply in Fort Worth, Dallas TX, USA) was used as the control diet and the base for the AP-supplemented diet, which Envigo, Corp. (Indianapolis, IN, USA) prepared. The AP-supplemented diet contained 0.014% AP. Based on the formula for this diet, a mouse with an average weight of 25–35 g would consume 20 mg/kg body weight (bw) of AP daily (referred to as AP 20). In practical terms, each mouse would consume 0.7 mg of AP per day. This calculation is derived from the average food consumption of a mouse, which is approximately 15 g of food per 100 g body weight per day [44,45]. Further, this AP 20 mg/kg bw daily (0.7 mg AP per a 35 mg mouse daily) is comparable with an intake of 0.667 mg AP daily by a 75 kg adult man (receiving one capsule of 50 mg AP dietary supplement daily). Figure 2 shows the diagram of the experimental design of the study.

Mice were placed on an AP diet consisting of either 0 mg/kg (AP 0) or 20 mg/kg (AP 20) of body weight (with or without irradiation). The rationale for using the AP 20 diet has been detailed in previous studies elsewhere [4,5]. For mice receiving 20 mg/kg bw (AP20 with or without ^28^Si-exposure), the AP diet began five days (−5d) before the ^28^Si-irradiation and continued until the mice reached 620 days of age. At that point, the weights of mice in each group had not changed significantly, and all mice that received AP 20 were switched to a regular diet (AP 0). Also, for those with the AP20 diet (with or without ^28^Si-irradiation), we chose a 5-day AP diet prior to ^28^Si-irradiation to ensure the bioavailability of AP for scavenging ^28^Si-induced ROS or RNS. Using this diet protocol, mice would receive the AP diet before and after exposure to ^28^Si ions, making it possible to investigate the protection, mitigation, and chemoprevention efficacies of AP, which is the primary goal of our project.

### 2.3. Whole-Body Exposure of Mice to 260 MeV/n, ^28^Si Ions

Groups of mice were whole-body exposed to 0 or 0.5 Gy of ^28^Si ions, which was carried out at the NASA Space Radiation Laboratory (NSRL) in Brookhaven National Laboratory (BNL), Upton, NY, USA, following previously published methods [4,46,47]. At the time this study was initiated, a single dose of 50 cGy of ^28^Si ions (typical for high doses of space radiation) was recommended by NASA, delivered at a dose rate of 50 cGy/min (personal communication). Overall, as shown in Figure 2, the study involved four groups of mice: **Group 1**: Sham controls (AP 0 No Rad), **Group 2**: Radiation only (AP 0 Rad), **Group 3**: AP diet only (AP 20 No Rad), and **Group 4**: Irradiated mice given the AP diet (AP 20 Rad). Figure 2 illustrates the experimental groups and the number of mice in each group. All experiment protocols were conducted under the approval of the Institutional Animal Care and Use Committee of both Stony Brook University (SBU) and BNL. The sham and AP diet only groups were exposed to the same stresses as the radiation groups.

All mice were monitored for morbidity and mortality until they reached about 770 d of age. The body weight of each mouse was obtained every six weeks until death. Mice that exhibited signs of being moribund were immediately euthanized. This allowed for the collection of tissues suitable for histological examination, enabling the accurate categorization of all neoplasms. Blood samples were collected for hematological evaluation and plasma preparation. The gross examination was performed at necropsy to identify potential lesions, nodules, or masses in several organs. If the lesions were detected in a specific organ, a portion of that specific tissue (plus other tissues, e.g., the liver, lung, kidney, spleen, and sternum) was removed and fixed in 10% buffered formalin, processed, embedded in paraffin wax, sectioned, and stained with hematoxylin and eosin for histopathological examination. Further, an immunohistochemically staining method with CD45 antibody (Cell Signaling Technology, Danvers, MA, USA, catalogue #70257 (Rabbit mAb)) was used to confirm the presence and type of leukemia. Of note, all histopathological evaluation were diagnosed under the guidelines of Mouse Models of Human Cancers Consortium Pathology Standing Committee for hematologic neoplasms [48].The resulting data would determine the effectiveness of AP in prevention, mitigation, or delay of acute lymphoma/lymphoblastic leukemia in exposed male C57BL/6 mice after exposure to 260 MeV/n ^28^Si ions as determined by incidence, aggressiveness, overall infiltration, and latency.

### 2.4. Statistical Analysis

The calculations concerning sample size and related power estimates for comparing two proportions used the procedures described in Equations (4.18) & (4.19) in J.L. Fleiss et al., *Statistical Methods for Rates and Proportions*, 4th ed. [49]. At the time of the initiation of the study, we used the incidence rate in the sham non-irradiated group (spontaneous rate) from existing literature, which was expected to be 7% of acute lymphoma/lymphoblastic leukemia at about 700–800 days of age. We used this rate as the foundation for the power analyses used to determine sample sizes in conjunction with projected (based on the literature and results from previous studies) radiation-related increases in incidence rates over background. All sample sizes for the comparisons were determined to meet or exceed an approximate 80% power. The effects of AP (20 mg/kg bw) in animals receiving no radiation were evaluated. Analyses of observed incidence rates (proportions) were made using Chi-Square tests for two groups. The Type I error rate was set at 0.05 and all tests for increases or decreases were one-sided. Kaplan–Meier curves were constructed, and a log-rank test was used to compare the survival of the groups. The results were considered significant if the *p*-value was less than 0.05. Analyses were facilitated using GraphPad Prism 10.

## 3. Results

### 3.1. Weights of Mice

**Figure 3:**
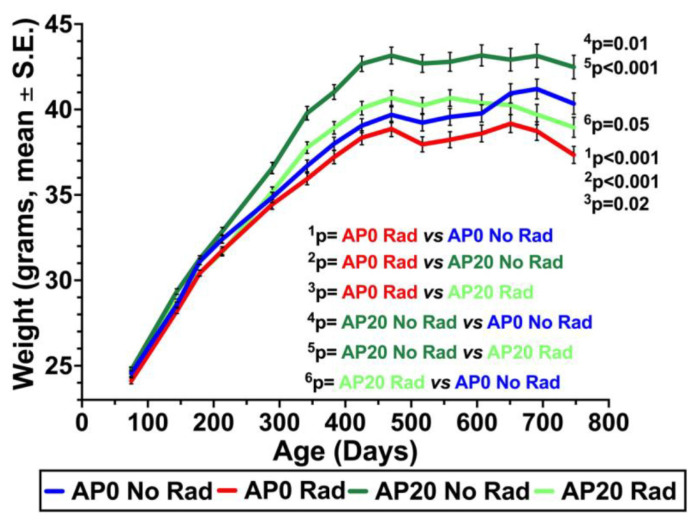
Temporal changes in the mean weight.

Figure 3 shows the temporal changes in the mean weight in each group. The weight of mice in each group increased precipitously with age. The highest rate of weight gain was detected in mice given the AP diet without irradiation (**dark green color**), while the lowest weight gain was found in irradiated mice without the AP diet (**red color**). The weight of irradiated mice with the AP diet (**light green color**) was less than those given the AP diet without irradiation, but higher than that of the control group (**blue color**) until about 625 days of age. In brief, mice with the AP diet (with or without irradiation) gain more weight than those without the AP diet. The trend of weight gain persisted until mice reached 475 days of age, at which point the weight started declining in irradiated mice without the AP diet. Overall, our findings suggest that the AP diet provides functional food and that daily consumption of AP is safe.

### 3.2. Survival Curve and Acute Lymphoma/Lymphoblastic Leukemia Incidence

**Figure 4:**
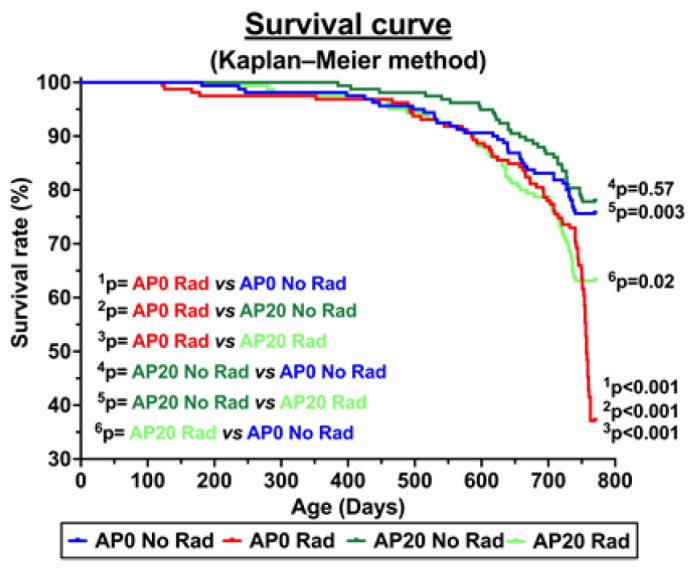
Survival curve of each group.

Kaplan–Meier curves were constructed, and a log-rank test was used to compare the survival of the groups. The results were considered significant if the *p*-value was less than 0.05. Figure 4 illustrates the survival curve of each group. Notably, 98% of the animals were euthanized while in a moribund state, preventing any that might have died unattended. In addition to the moribund state (i.e., lethargy, hunched posture, reluctance to move, hunched posture, closed eyes, signs of distress, difficulty breathing), some mice with severe ulcerative dermatitis were also euthanized and necropsied. Not all moribund mice were caused by acute lymphoma/lymphoblastic leukemia.

**Figure 5:**
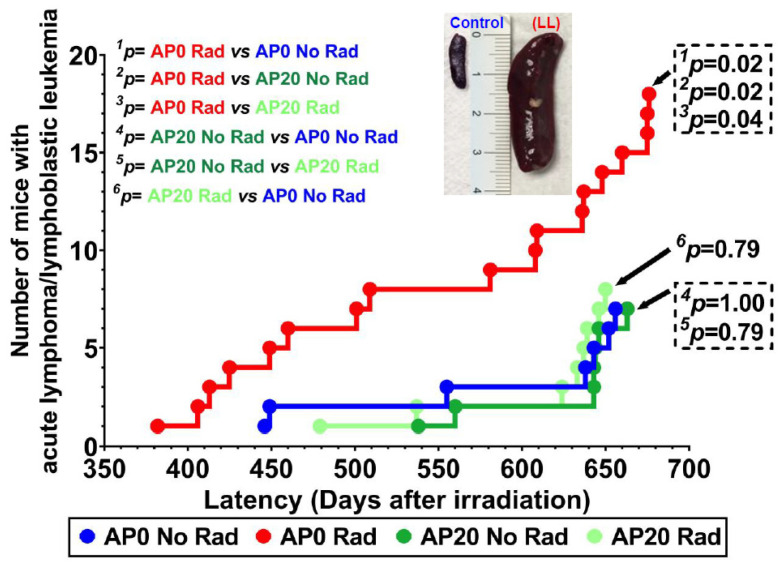
Incidence of acute lymphoma/lymphoblastic leukemia (LL).

At 770 days of age, the lowest survival rate was found in the ^28^Si-irradiated mice (37%), but AP consumption improved the survival rate in ^28^Si-irradiated mice (63%). Although there is a trend of more surviving mice in those with AP diet, as compared to the control group, there is no statistical difference in survival between these two groups (*p* = 0.57). Our data also indicate that, starting around 250 days of age, the survival rates of irradiated mice with and without an AP diet were similar. However, a significant difference in survival rates between these two groups was observed at 770 days (*p* < 0.001, ^3^p in Figure 5), indicating the protective effects of the AP diet.

Notably, over 98% of the animals were euthanized while in a moribund state, preventing any that might have died unattended. Hence, 98% of the animals in the study underwent prompt necropsies, ensuring the collection of tissues that were suitable for histopathological examination. This process allowed for the clear categorization of all neoplasms. Figure 5 shows the incidence of acute lymphoma/lymphoblastic leukemia in each experimental group. We found 18 cases of acute lymphoma/lymphoblastic leukemia in ^28^Si-irradiated mice without an AP diet (**AP 0 Rad**, red color), while there were 7 cases in the control group (**AP 0 No Rad**, blue color). We also found 7 cases in mice fed an AP diet without ^28^Si-irradiation (**AP 20 No Rad**, dark green color), whereas 8 cases of acute lymphoma/lymphoblastic leukemia were found in ^28^Si-irradiated mice fed an AP diet (**AP 20 Rad**, light green color). Hence, a 2.57-fold increase in the incidence of acute lymphoma/lymphoblastic leukemia was observed in irradiated mice without an AP diet, compared to the control group or to non-irradiated mice fed the AP diet (*p* = 0.02); while a 2.25-fold increase compared to irradiated mice fed the AP diet, (*p* = 0.04) was found. No statistical differences were observed in the incidence of acute lymphoma/lymphoblastic leukemia among the control group, mice fed an AP diet without irradiation, and irradiated mice on an AP diet, which reported 7, 7, and 8 cases, respectively.

The first case of acute lymphoma/lymphoblastic leukemia in this study was detected in the irradiated mice that were not on the AP diet, specifically 382 days after irradiation. The first case in the control group appeared at 446 days post-irradiation, while the first cases of acute lymphoma/lymphoblastic leukemia in irradiated mice on the AP diet and non-irradiated mice on the AP diet were identified at 479 and 536 days after irradiation, respectively. Notably, the appearance of acute lymphoma/lymphoblastic leukemia cases in irradiated mice without an AP diet was detected throughout the study; however, most cases in other groups were found between 640 to 660 days post-irradiation.

Table 1, Table 2, Table 3 and Table 4 present the hematological and histopathological data for each case across the different groups. These tables include information for the control group (AP0 No Rad), irradiated mice without the AP diet (AP0 Rad), non-irradiated mice that received the AP diet (AP20 No Rad), and irradiated mice on the AP diet (AP20 Rad). Additionally, each table details the infiltration of acute lymphoma/lymphoblastic leukemia (referred to as LL) into various organs in each case. It is important to note that two cases lack hematological data because the animals were found dead: one from the control group (Table 1, case #3, found dead at 555 days post-irradiation) and one from the irradiated group on the AP diet (Table 4, case #3, found dead at 624 days post-irradiation). Furthermore, the white blood cell (WBC) count in one case from the non-irradiated mice fed the AP diet (Table 3, case #3) was “out of range high,” leading to elevated levels of neutrophils (NEUT) and monocytes (MONO) for that mouse. The cause of these parameters being outside the statistically determined reference interval for healthy mice remains unknown.

Notably, this case of acute lymphoma/lymphoblastic leukemia infiltrated four organs: the liver, lung, spleen, and thymus, while other cases affected only two or three organs. The spleen was the primary affected organ, followed by the thymus, liver, lung, and kidneys. Enlarged spleens were observed in all cases, with an average size that was 2.5 to 3 times larger in mice with acute lymphoma/lymphoblastic leukemia compared to control mice of similar age, as illustrated in the inset of Figure 5.

Table 5 shows the mean ± standard error (S.E.) values of major hematological parameters (i.e., RBC, PLT, WBC, LYMPH, NEUT, and MONO) for acute lymphoma/lymphoblastic leukemia cases in each group. Our data indicate that the mean values of RBC, PLT, and LYMPH for these cases, regardless of the experimental group, were lower than those of normal male C57BL/6J mice. In contrast, the mean values of WBC, NEUT, and MONO for acute lymphoma/lymphoblastic leukemia cases were higher compared to normal male C57BL/6J mice.

**Figure 6:**
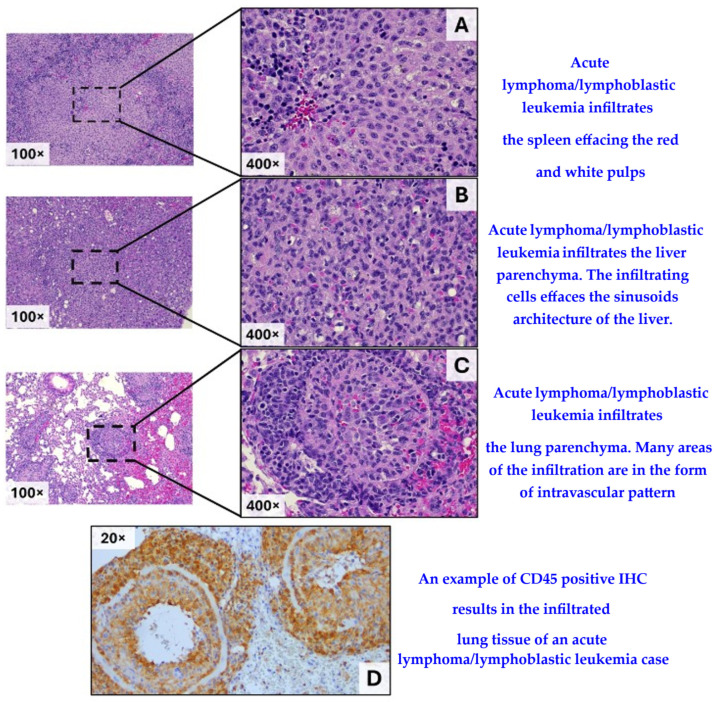
Histopathological evidence of infiltration by acute lymphoma/lymphoblastic leukemia cells into various organs ((**A**), (**B**), (**C**) by H&E staining in the spleen, the liver, and the lung, respectively; while (**D)** is an example of CD45 positive IHC results in the infiltrated lung tissue of an acute lymphoma/lymphoblastic case).

Figure 6 shows examples of histological evidence of infiltration by acute lymphoma/lymphoblastic leukemia cells into various organs of the affected mice.

## 4. Discussion

Our study is the first to test the administration of AP to mice as a dietary supplement for preventing and mitigating the induction of acute lymphoma/lymphoblastic leukemia from ^28^Si-exposure. The data presented here is part of a larger study examining the effectiveness of AP as a countermeasure against both early and late harmful effects resulting from exposure to 0.5 Gy of 260 MeV/n ^28^Si ions, which are important components of space radiation. In previous reports, we highlighted the effectiveness of AP in protecting bone marrow cells [4] and gut tissue [5] collected seven days post-irradiation from groups of mice that are part of the same cohort as this study. In the current research, our primary focus is to evaluate the use of AP as a dietary supplement to prevent acute lymphoma/lymphoblastic leukemia in male C57BL/6 mice exposed to 0.5 Gy of 260 MeV/n ^28^Si ions. We also assessed tumor infiltration and latency of acute lymphoma/lymphoblastic leukemia in these mice.

Our results indicate that daily feeding of an AP diet at a concentration of 20 mg/kg bw prevents the induction of acute lymphoma/lymphoblastic leukemia in the exposed male C57BL/6 mice. Mice exposed to ^28^Si ions without the AP diet exhibited a 2.25-fold increase in incidence compared to control mice. In contrast, mice exposed to ^28^Si ions while fed the AP diet, as well as non-irradiated mice receiving the AP diet, did not exhibit this increase. No statistical differences were observed in the incidence of acute lymphoma/lymphoblastic leukemia among control mice, non-exposed mice fed the AP diet, and exposed mice that were also fed the AP diet. These findings demonstrate the effectiveness of the AP diet in preventing or mitigating the effects of ^28^Si-ion-induced acute lymphoma/lymphoblastic leukemia. Overall, our results not only demonstrate the protective qualities of AP against ^28^Si-ion-induced acute lymphoma/lymphoblastic leukemia but also emphasize that AP is easily administered (via the oral route), making it suitable for self-administration and dosing for human in space.

The shortest latency period for acute lymphoma/lymphoblastic leukemia occurred in irradiated mice that did not receive the AP diet (382 days post-irradiation), while the longest latency period was observed in non-irradiated mice on the AP diet (536 days post-irradiation). Our findings indicate that acute lymphoma/lymphoblastic leukemia cases from irradiated mice without an AP diet progressed more quickly than cases from other groups. This may be due to the higher levels of initial damage in the hematopoietic tissues caused by ^28^Si ions (e.g., oxidative damage leading to inflammation) in exposed mice not fed the AP diet, compared to those that were irradiated but fed the AP diet, resulting in a slower progression of the disease in the latter group. Such initial damage can progress into chronic oxidative damage and chronic inflammation. These findings reinforce the hypothesis that the AP diet confers protective and mitigative effects on space radiation damage. The longer latency of acute lymphoma/lymphoblastic leukemia in non-irradiated mice fed the AP diet (536 days post-irradiation) compared to the control group (446 days post-irradiation) demonstrates the health benefits of the AP diet. Moreover, our results showed a higher tumor infiltration in acute lymphoma/lymphoblastic leukemia cases induced by ^28^Si ions compared to other groups, suggesting that the cancer was more severe in these mice, as compared to those from other experimental groups. Notably, we identified only one case of acute lymphoma/lymphoblastic leukemia in non-irradiated mice fed the AP diet, with tumor infiltrations observed in four organs: the spleen, thymus, liver, and lung of the affected mouse. The mechanisms underlying the aggressiveness of this acute lymphoma/lymphoblastic leukemia case remain unknown. Overall, our findings suggest that dietary AP is an effective countermeasure against acute lymphoma/lymphoblastic leukemia induced by ^28^Si ions.

As previously mentioned, we investigated the effects of the AP diet as a countermeasure against early damage caused by ^28^Si-ion exposure in bone marrow (BM) cells and the gut tissue (duodenum) collected at d 7 post-irradiation from groups of mice that were the same cohort used to investigate the incidence of acute lymphoma/lymphoblastic leukemia. Our results demonstrate that the AP diet can both prevent and mitigate ^28^Si-induced inflammation, as indicated by elevated levels of activated NF-κB and pro-inflammatory cytokines in both types of tissue collected from the same mouse [4]. This finding highlights the effectiveness of the AP diet in suppressing inflammation induced by ^28^Si ions across different tissues. Such a capability is one of the key characteristics of an ideal radiation countermeasure.

Results from several ground-based animal studies conducted in our [47,50,51,52] and other laboratories [53,54,55,56,57,58] have shown that space radiation induces acute oxidative damage and acute inflammation, leading to chronic oxidative damage and inflammation in various tissues of exposed mice, partly due to the feedback mechanism [47,50,59]. Such findings are critically important because it has been well recognized that persistent oxidative damage and inflammation are highly relevant to cancer induction [60]. Previously, we reported that ^28^Si-exposure induces persistently high levels of activated NF-κB and related pro-inflammatory cytokines in the BM tissue of exposed CBA/Ca mice [47], suggesting that exposure to ^28^Si ions induces chronic inflammation, regardless of the mouse strain. Furthermore, we observed a connection between chronic inflammation and the in vivo induction of late-occurring chromosomal damage (genomic instability) [61], which is known to be a key event in cancer induction [60,62].

Although this study does not provide data on changes over time in the levels of activated NF-κB and NF-κB-related pro-inflammatory cytokines, the AP diet had significant counteracting effects at an early stage. This highlights its potential as a preventive measure against chronic inflammation, which may lead to the prevention of acute lymphoma and lymphoblastic leukemia. Further, our findings show that the AP diet can both prevent and mitigate inflammation induced by ^28^Si (as evidenced by elevated levels of activated NF-κB and pro-inflammatory cytokines) in the BM and the gut tissues collected from the same mouse, which is part of the same mouse cohort included in this cancer bioassay. Therefore, it is reasonable to propose that AP protects mice from ^28^Si-induced acute lymphoma/lymphoblastic leukemia through its anti-inflammatory activity. Notably, our previous study on the early harmful effects of ^28^Si ions [4] also demonstrated that the AP diet prevents leukopenia and thrombocytopenia in these mice, illustrating the countermeasure effectiveness of AP against radiation-induced injuries in the hematopoietic system. Furthermore, and quite intriguingly, in the same mouse, we found a decrease in the abundance of beneficial (probiotic) bacteria (e.g., *Lachnospiraceae*, *Muribaculaceae*, and *Bifidobacteriaceae*) in the irradiated mice without the AP diet, and an increase in inflammation in the gut tissue (as evident by an increased level of activated NF-κB and pro-inflammatory cytokines). Of note, it has been reported that the reduced abundance of these beneficial bacteria is associated not only with immune dysfunction but also defects in the integrity of the local epithelial barrier [63,64], due to low levels of single-chain fatty acids (SCFAs). In contrast, reduced inflammation, less reduction in the beneficial bacterial populations, and a restoration of normal gut histological structure were observed in ^28^Si-exposed mice that received the AP diet, suggesting that AP acts as a countermeasure by both its anti-inflammatory actions and through a favorable impact on the gut microbiome [5].

Exciting new research is uncovering a connection between gut health and overall well-being. An imbalanced gut microbiome, often referred to as dysbiosis, has been increasingly linked to serious conditions such as inflammation and cancer [65,66] and many other diseases [67]. This critical connection underscores the importance of maintaining a healthy gut for overall good health. Growing evidence suggests that the gut–bone marrow axis significantly influences radiation-induced acute lymphoma/lymphoblastic leukemia, particularly through an impact of the gut microbiome on the bone marrow (BM) microenvironment and inflammatory responses [68,69]. Therefore, it is plausible to suggest that AP exerts its countermeasure effects not only through a direct impact on the marrow and gut, but also via the “gut–bone marrow axis”.

Our results further indicate that long-term consumption of the AP diet (20 mg/kg body weight of the C57BL/6 mice) is safe, reinforcing the existing safety profile of AP, which is generally regarded as safe and shows no severe toxicity, even at higher doses. Studies assessing the acute toxicity of AP have reported no mortality or signs of toxicity in mice or rats given oral doses of up to 5000 mg/kg [70]. In this study, we measured plasma levels of the clinical “liver function test” enzymes, aspartate aminotransferase (AST) and alanine aminotransferase (ALT) in a few mice from each group at one year of age. We found no increases in these enzymes, which suggests no liver damage (personal communication) from daily consumption of the AP diet at the concentration of 20 mg/kg bw in male C57BL/6 mice.

Relating to the survival curve, our data demonstrate a typical survival curve associated with radiation exposure or aging effects. Specifically, we observe an initial “shoulder” phase, followed by an exponential decline in survival as the dose of radiation or age increases. It is well established that radiation accelerates aging-like effects, shortens lifespan, and increases the risk of cancer. Additionally, mutations that affect the aging process can also influence an organism’s resistance to radiation. These two processes are interconnected through mechanisms such as DNA damage, oxidative stress, and inflammation which accumulate over time. Notably, the survival rate shows a steep decline around 720 days of age in irradiated mice that did not receive the AP diet. In contrast, the survival curve also illustrates the protective effects of the AP diet in irradiated mice that were fed this diet.

## 5. Conclusions

Our results demonstrate the potential of dietary AP as a countermeasure against Si-ion-induced acute lymphoma/lymphoblastic leukemia, making oral AP a viable and innovative candidate for mitigating the effects of space radiation. Together with our findings of the effectiveness of AP in counteracting the early effects of ^28^Si exposure in mice from the same cohorts in this long-term research, we propose that the gut–bone marrow axis influences how the immune system responds to ^28^Si ions and affects the development of acute lymphoma and lymphoblastic leukemia. While our study has yielded significant findings, certain limitations should be acknowledged. We used only ^28^Si ions, which do not fully represent the space environment, which includes various types of heavy ions and proton-dominated solar particle events. At the time this study began, NASA’s ground-based Galactic Cosmic Ray Simulator (GCRSim), capable of generating multiple beams simultaneously, was not available. In future research on medical countermeasures, it is essential to utilize the GCRSim. This study only included male mice. Future studies should also involve female mice, as research has shown that there are gender-specific differences in response to radiation. Additionally, having data on chronic oxidative damage, chronic inflammation, and levels of short-chain fatty acids (SCFAs) would enhance our understanding of the role of the gut–bone marrow axis in the induction of acute lymphoma/lymphoblastic leukemia following radiation exposure. Such knowledge would play an important role in the development of a more effective therapeutic strategy. Importantly, clinical trials are necessary to confirm their efficacy and safety in humans.

## Figures and Tables

**Table 1:** Hematological parameters and histopathological evaluation of acute lymphoma/lymphoblastic leukemia cases from the control group (AP 0 No Rad).

Case #	Days, Post Irradiation (Latency)	Complete Blood Count with Differential Count	Histopathological Evaluation							
			Bone	Gut	Kidney	Liver	Lung	Pancreas	Spleen	Thymus
1	445	**CBC** RBC = 2.94 × 10^6^ cells/µL PLT = 840 × 10^3^ cells/µL WBC = 23.75 × 10^3^ cells/µL **Differential count** nd	na	na	na	LL	npd	na	LL	LL
2	449	**CBC** RBC = 4.74 × 10^6^ cells/µL PLT = 540 × 10^3^ cells/µL WBC = 43.35 × 10^3^ cells/µL **Differential count** nd	no	na	na	npd	LL	na	npd	LL
3	555	**CBC** nd	no	na	npd	npd	npd	na	npd	LL
4	638	**CBC** RBC = 6.51 × 10^6^ cells/µL PLT = 167 × 10^3^ cells/µL WBC = 2.82 × 10^3^ cells/µL **Differential count** NEUT = 1.21 × 10^3^ cells/µL LYMPH = 1.03 × 10^3^ cells/µL MONO = 0.19 × 10^3^ cells/µL EOS = 0.31 × 10^3^ cells/µL BASO = 0.07 × 10^3^ cells/µL	no	npd	npd	LL	LL	npd	LL	na
5	643	**CBC** RBC = 8.01 × 10^6^ cells/µL PLT = 1251 × 10^3^ cells/µL WBC = 5.42 × 10^3^ cells/µL **Differential count** NEUT = 1.39 × 10^3^ cells/µL LYMPH = 1.91 × 10^3^ cells/µL MONO = 0.57 × 10^3^ cells/µL EOS = 0.86 × 10^3^ cells/µL BASO = 0.68 × 10^3^ cells/µL	no	na	npd	npd	npd	na	LL	na
6	652	**CBC** RBC = 6.35 × 10^6^ cells/µL PLT = 1067 × 10^3^ cells/µL WBC = 5.14 × 10^3^ cells/µL **Differential count** NEUT = 1.62 × 10^3^ cells/µL LYMPH = 1.9 × 10^3^ cells/µL MONO = 0.43 × 10^3^ cells/µL EOS = 0.82 × 10^3^ cells/µL BASO = 0.37 × 10^3^ cells/µL	no	na	npd	npd	npd	na	LL	LL
7	656	**CBC** RBC = 6.54 × 10^6^ cells/µL PLT = 1229 × 10^3^ cells/µL WBC = 4.92 × 10^3^ cells/µL **Differential count** NEUT = 1.64 × 10^3^ cells/µL LYMPH = 1.58 × 10^3^ cells/µL MONO = 0.2 × 10^3^ cells/µL EOS = 0.36 × 10^3^ cells/µL BASO = 1.14 × 10^3^ cells/µL	na	npd	npd	npd	LL	npd	npd	LL

npd = no pathological diagnosis; na = not available; no = not optimal staining; LL = acute lymphoma/lymphoblastic leukemia; nd = not done.

**Table 2:** Hematological parameters and histopathological evaluation of acute lymphoma/lymphoblastic leukemia cases from mice exposed to ^28^Si ions without the AP diet (AP 0 Rad).

Case #	Days, Post Irradiation (Latency)	Complete Blood Count with Differential Count	Histopathological Evaluation							
			Bone	Gut	Kidney	Liver	Lung	Pancreas	Spleen	Thymus
1	382	**CBC** RBC = 15.65 × 10^6^ cells/µL PLT = uncountable WBC = uncountable **Differential count** nd	no	npd necrosis	npd	LL	npd	na	LL	na
2	406	**CBC** RBC = 5.98 × 10^6^ cells/µL PLT = 180 × 10^3^ cells/µL WBC = 15.4 × 10^3^ cells/µL **Differential count** nd	no	npd	na	LL	LL	npd	LL	na
3	413	**CBC** RBC = 8.3 × 10^6^ cells/µL PLT = 480 × 10^3^ cells/µL WBC = 3.35 × 10^3^ cells/µL **Differential count** nd	na	na	npd	npd	npd	na	npd	LL
4	425	**CBC** RBC = 3.66 × 10^6^ cells/µL PLT = 120 × 10^3^ cells/µL WBC = 31.45 × 10^3^ cells/µL **Differential count** nd	no	na	npd	LL	npd	na	LL	na
5	449	**CBC** RBC = 2.28 × 10^6^ cells/µL PLT =270 × 10^3^ cells/µL WBC = 295.5 × 10^3^ cells/µL **Differential count** nd	no	npd	npd	npd	npd	na	LL	na
6	460	**CBC** RBC = 3.26 × 10^6^ cells/µL PLT = 320 × 10^3^ cells/µL WBC = 226.75 × 10^3^ cells/µL **Differential count** nd	no	npd	npd	LL	LL	na	LL	na
7	501	**CBC** RBC = 2.76 × 10^6^ cells/µL PLT = 20 × 10^3^ cells/µL WBC = 518 × 10^3^ cells/µL **Differential count** nd	no	na	npd	LL	npd	na	LL	na
8	509	**CBC** RBC = 1.28 × 10^6^ cells/µL PLT = 40 × 10^3^ cells/µL WBC = 292 × 10^3^ cells/µL **Differential count** nd	no	npd	npd	fatty liver	npd	npd	LL	na
9	581	**CBC** RBC = 5.52 × 10^6^ cells/µL PLT = 875 × 10^3^ cells/µL WBC = 4.92 × 10^3^ cells/µL **Differential count** nd	no	na	npd	npd	npd	na	npd	LL
10	608	**CBC** RBC = 3.35 × 10^6^ cells/µL PLT = 100 × 10^3^ cells/µL WBC = 6.28 × 10^3^ cells/µL **Differential count** NEUT = 3.21 × 10^3^ cells/µL LYMPH = 2.12 × 10^3^ cells/µL MONO = 0.7 × 10^3^ cells/µL EOS = 0.16 × 10^3^ cells/µL BASO = 0.09 × 10^3^ cells/µL	no	npd necrosis	npd	LL	npd	npd	lpd	na
11	609	**CBC** RBC = 7.01 × 10^6^ cells/µL PLT = 1741 × 10^3^ cells/µL WBC = 3.88 × 10^3^ cells/µL **Differential count** NEUT = 1.64 × 10^3^ cells/µL LYMPH = 1.58 × 10^3^ cells/µL MONO = 0.2 × 10^3^ cells/µL EOS = 0.36 × 10^3^ cells/µL BASO = 1.14 × 10^3^ cells/µL	na	na	npd	npd	npd	na	npd	LL
12	636	**CBC** RBC = 7.7 × 10^6^ cells/µL PLT = 1741 × 10^3^ cells/µL WBC = 3.88 × 10^3^ cells/µL **Differential count** NEUT = 1 × 10^3^ cells/µL LYMPH = 1.37 × 10^3^ cells/µL MONO = 0.35 × 10^3^ cells/µL EOS = 0.66 × 10^3^ cells/µL BASO = 0.5 × 10^3^ cells/µL	no	na	npd	LL	npd	na	LL	LL
13	637	**CBC** RBC = 2.94 × 10^6^ cells/µL PLT = 503 × 10^3^ cells/µL WBC = 8.94 × 10^3^ cells/µL **Differential count** NEUT = 4.34 × 10^3^ cells/µL LYMPH = 3.04 × 10^3^ cells/µL MONO = 0.67 × 10^3^ cells/µL EOS = 0.72 × 10^3^ cells/µL BASO = 0.16 × 10^3^ cells/µL	no	npd	npd	LL	npd	npd	npd	na
14	648	**CBC** RBC = 3.52 × 10^6^ cells/µL PLT = 376 × 10^3^ cells/µL WBC = 1.54 × 10^3^ cells/µL **Differential count** NEUT = 0.52 × 10^3^ cells/µL LYMPH = 0.56 × 10^3^ cells/µL MONO = 0.17 × 10^3^ cells/µL EOS = 0.26 × 10^3^ cells/µL BASO = 0.03 × 10^3^ cells/µL	no	npd	npd	LL	LL	npd	LL	na
15	660	**CBC** RBC = 3.95 × 10^6^ cells/µL PLT = 1178 × 10^3^ cells/µL WBC = 4.12 × 10^3^ cells/µL **Differential count** NEUT = 1.07 × 10^3^ cells/µL LYMPH = 0.88 × 10^3^ cells/µL MONO = 0.35 × 10^3^ cells/µL EOS = 0.96 × 10^3^ cells/µL BASO = 0.86 × 10^3^ cells/µL	no	npd	npd	npd	npd	npd	LL	LL
16	675	**CBC** RBC = 8.47 × 10^6^ cells/µL PLT = 1872 × 10^3^ cells/µL WBC = 11.28 × 10^3^ cells/µL **Differential count** NEUT = 3.25 × 10^3^ cells/µL LYMPH = 5.6 × 10^3^ cells/µL MONO = 0.95 × 10^3^ cells/µL EOS = 0.95 × 10^3^ cells/µL BASO = 0.53 × 10^3^ cells/µL	no	npd	npd	liver tumor	npd	na	LL	na
17	675	**CBC** RBC = 8.41 × 10^6^ cells/µL PLT = 1872 × 10^3^ cells/µL WBC = 10.82 × 10^3^ cells/µL **Differential count** NEUT = 3.21 × 10^3^ cells/µL LYMPH = 4.71 × 10^3^ cells/µL MONO = 1.13 × 10^3^ cells/µL EOS = 1.33 × 10^3^ cells/µL BASO = 0.43 × 10^3^ cells/µL	no	na	npd	fatty liver	npd	na	LL	LL
18	676	**CBC** RBC = 7.42 × 10^6^ cells/µL PLT = 1825 × 10^3^ cells/µL WBC = 12.04 × 10^3^ cells/µL **Differential count** NEUT = 2.64 × 10^3^ cells/µL LYMPH = 6.05 × 10^3^ cells/µL MONO = 1.12 × 10^3^ cells/µL EOS = 1.67 × 10^3^ cells/µL BASO = 0.56 × 10^3^ cells/µL	no	npd	npd	npd	LL	npd	LL	na

npd = no pathological diagnosis; na = not available; no = not optimal staining; LL = acute lymphoma/lymphoblastic leukemia; lpd = lymphoproliferative disorder; nd = not done.

**Table 3:** Hematological parameters and histopathological evaluation of acute lymphoma/lymphoblastic leukemia cases from non-irradiated mice fed with the AP diet (AP 20 No Rad).

Case #	Days, Post Irradiation (Latency)	Complete Blood Count with Differential Count	Histopathological Evaluation							
			Bone	Gut	Kidney	Liver	Lung	Pancreas	Spleen	Thymus
1	538	**CBC** RBC = 2.22 × 10^6^ cells/µL PLT = 460 × 10^3^ cells/µL WBC = 161.2 × 10^3^ cells/µL **Differential count** nd	na	npd	npd	LL	npd	npd	lpd	na
2	560	**CBC** RBC = 3.6 × 10^6^ cells/µL PLT = 350 × 10^3^ cells/µL WBC = 480 × 10^3^ cells/µL **Differential count** nd	na	npd	npd	LL	LL	npd	lpd	na
3	643	**CBC** RBC = 6.15 × 10^6^ cells/µL PLT = 1178 × 10^3^ cells/µL WBC = high **Differential count** NEUT = high LYMPH = high MONO = 69.44 × 10^3^ cells/µL EOS = 68.24 × 10^3^ cells/µL BASO = 21.78 × 10^3^ cells/µL	no	na	npd	LL	LL	na	LL	LL
4	643	**CBC** RBC = 4.77 × 10^6^ cells/µL PLT = 180 × 10^3^ cells/µL WBC = 11.76 × 10^3^ cells/µL **Differential count** NEUT = 7.2 × 10^3^ cells/µL LYMPH = 2.96 × 10^3^ cells/µL MONO = 0.84 × 10^3^ cells/µL EOS = 0.8 × 10^3^ cells/µL BASO = 0.11 × 10^3^ cells/µL	no	npd	npd	LL	LL	npd	LL	na
5	644	**CBC** RBC = 6.25 × 10^6^ cells/µL PLT = 2365 × 10^3^ cells/µL WBC = 6.36 × 10^3^ cells/µL **Differential count** NEUT = 1.79 × 10^3^ cells/µL LYMPH = 2.77 × 10^3^ cells/µL MONO = 0.47 × 10^3^ cells/µL EOS = 0.7 × 10^3^ cells/µL BASO = 0.43 × 10^3^ cells/µL	no	na	npd	npd	npd	na	LL	LL
6	646	**CBC** RBC = 9.09 × 10^6^ cells/µL PLT = 1069 × 10^3^ cells/µL WBC = 5.38 × 10^3^ cells/µL **Differential count** NEUT = 1.53 × 10^3^ cells/µL LYMPH = 2.54 × 10^3^ cells/µL MONO = 0.47 × 10^3^ cells/µL EOS = 0.58 × 10^3^ cells/µL BASO = 0.26 × 10^3^ cells/µL	no	na	npd	LL	npd	na	LL	na
7	663	**CBC** RBC = 5.8 × 10^6^ cells/µL PLT = 879 × 10^3^ cells/µL WBC = 3.43 × 10^3^ cells/µL **Differential count** NEUT = 1.57 × 10^3^ cells/µL LYMPH = 1.06 × 10^3^ cells/µL MONO = 0.25 × 10^3^ cells/µL EOS = 0.96 × 10^3^ cells/µL BASO = 0.5 × 10^3^ cells/µL	no	npd	nod	npd	npd	na	LL	LL

npd = no pathological diagnosis; na = not available; no = not optimal staining; LL = acute lymphoma/lymphoblastic leukemia; lpd = lymphoproliferative disorder; nd = not done.

**Table 4:** Hematological parameters and histopathological evaluation of acute lymphoma/lymphoblastic leukemia cases from ^28^Si-irradaited mice fed with the AP diet (AP 20 Rad).

Case #	Days, Post Irradiation (Latency)	Complete Blood Count with Differential Count	Histopathological Evaluation							
			Bone	Gut	Kidney	Liver	Lung	Pancreas	Spleen	Thymus
1	479	**CBC** RBC = 3.84 × 10^6^ cells/µL PLT = 300 × 10^3^ cells/µL WBC = 182 × 10^3^ cells/µL **Differential count** nd	no	npd	LL	necrosis	na	npd	LL	LL
2	537	**CBC** RBC = 4.075 × 10^6^ cells/µL PLT = 1475 × 10^3^ cells/µL WBC = 245 × 10^3^ cells/µL **Differential count** nd	no	na	npd	npd	npd	npd	npd	LL
3	624	**CBC** nd **Differential count** nd	no	npd	npd	LL	LL	na	LL	na
4	633	**CBC** RBC = 6.86 × 10^6^ cells/µL PLT = 1965 × 10^3^ cells/µL WBC = 3.24 × 10^3^ cells/µL **Differential count** NEUT = 0.89 × 10^3^ cells/µL LYMPH = 1.26 × 10^3^ cells/µL MONO = 0.21 × 10^3^ cells/µL EOS = 0.8 × 10^3^ cells/µL BASO = 0.11 × 10^3^ cells/µL	no	na	npd	na	npd	na	LL	LL
5	637	**CBC** RBC = 6.25 × 10^6^ cells/µL PLT = 2365 × 10^3^ cells/µL WBC = 6.36 × 10^3^ cells/µL **Differential count** NEUT = 0.96 × 10^3^ cells/µL LYMPH = 1.48 × 10^3^ cells/µL MONO = 0.34 × 10^3^ cells/µL EOS = 0.91 × 10^3^ cells/µL BASO = 0.66 × 10^3^ cells/µL	no	na	npd	necrosis	npd	na	npd	LL
6	639	**CBC** RBC = 5.03 × 10^6^ cells/µL PLT = 1195 × 10^3^ cells/µL WBC = 8.96 × 10^3^ cells/µL **Differential count** NEUT = 3.31 × 10^3^ cells/µL LYMPH = 3.61 × 10^3^ cells/µL MONO = 0.7 × 10^3^ cells/µL EOS = 0.97 × 10^3^ cells/µL BASO = 0.36 × 10^3^ cells/µL	no	na	npd	LL	npd	na	LL	LL
7	646	**CBC** RBC = 5.25 × 10^6^ cells/µL PLT = 1007 × 10^3^ cells/µL WBC = 11.08 × 10^3^ cells/µL **Differential count** NEUT = 3.75 × 10^3^ cells/µL LYMPH = 4.48 × 10^3^ cells/µL MONO = 0.38 × 10^3^ cells/µL EOS = 1.24 × 10^3^ cells/µL BASO = 1.23 × 10^3^ cells/µL	no	npd	npd	npd	npd	npd	npd	LL
8	650	**CBC** RBC = 8.14 × 10^6^ cells/µL PLT = 996 × 10^3^ cells/µL WBC = 10.08 × 10^3^ cells/µL **Differential count** NEUT = 4.11 × 10^3^ cells/µL LYMPH = 1.92 × 10^3^ cells/µL MONO = 0.51 × 10^3^ cells/µL EOS = 3.06 × 10^3^ cells/µL BASO = 0.48 × 10^3^ cells/µL	no	na	npd	necrosis	na	na	LL	LL

npd = no pathological diagnosis; na = not available; no = not optimal staining; LL = acute lymphoma/lymphoblastic leukemia; nd = not done.

**Table 5:** Summary of the counts of major hematological parameters (Mean ± S.E.) of acute lymphoma/lymphoblastic leukemia cases in each group.

Treatment Group	RBC (×10^6^ Cells/µL)	PLT (×10^3^ Cells/µL)	WBC (×10^3^ Cells/µL)	LYMPH (×10^3^ Cells/µL)	NEUT (×10^3^ Cells/µL)	MONO (×10^3^ Cells/µL)
**AP 0 No Rad**	5.85 ± 0.72	849 ± 174.57	14.23 ± 6.62	1.61 ± 0.21	1.47 ± 0.10	0.35 ± 0.09
**AP 0 Rad**	5.64 ± 0.81	77.94 ± 182.15	85.30 ± 36.90	2.88 ± 0.69	2.32 ± 0.44	0.63 ±0.13
**AP 20 No Rad**	5.41 ± 0.83	925 ± 278.70	111.36 ± 77.93	2.33 ± 0.43	3.02 ± 0.43	14.29 ±13.79
**AP 20 Rad**	5.64 ± 0.58	1329 ± 257.76	38.54 ± 38.54	2.49 ± 0.61	2.66 ± 0.71	0.43 ± 0.08

Note: The counts of these parameters from normal male C57BL6/J are as follows (https://media.jax.org/m/4557d3ecbf2864af/original/033076_physiological_data.pdf (accessed on 18 October 2025)): 10.9 ± 0.2 × 10^6^ cells/µL for RBC; 1347 ± 168 × 10^3^ cells/µL for PLT; 8.4 ± 1.7 × 10^3^ cells/µL for WBC; 7.4 ±1.5 × 10^3^ cells/µL for LYMPH; 0.6 ± 0.1 × 10^3^ cells/µL for NEUT; and 0.17 ± 0.06 × 10^3^ cells/µL for MONO.

## Data Availability

Data are presented in the article.
